# 

**Published:** 1865-02

**Authors:** 


### CHICAGO MEDICAL JOURNAL.

**Terms, $2.00 per Annum.**

### CONTENTS OF THE FEBRUARY NUMBER.

PAGE.

ORIGINAL COMMUNICATIONS.

Transmission of Hereditary Diseases.—Animal Grafting—Origin of Tubercular Matter—Solidist's Theory of It. By E. Nichols, M. D. 49

Fœtal Hydrocephalus. By C. A. White, M. D. .......... 55

Fogyism in Physic. By W. C. Munson, M. D. .......... 57

Treatment of Tape-Worm with Elm Bark. By J. R. Dowler, M. D., of Beardstown, Ill. .......... 87

SELECTED.

On Hospital Gangrene, and Its Efficient Treatment. By John H. Packard, M. D., of Philadelphia .......... 68

Permanganate of Potash as a Remedy for Diphtheria. By Louis Mackall, jr., M. D., Georgetown, D. C. .......... 75

Effects of Tropical Climates on the European Constitution .......... 76

On the Pathology of Deposits of Urates. By Dr. George Harley, Prof. in University College, and Asst.-Physician to University College Hospital .......... 81

Counter-Irritation with Tincture of Iodine in Neuralgia .......... 91

Origin of Brandy .......... 94

EDITORIAL AND MISCELLANEOUS.

Rush Medical College .......... 84

